# Comparison of the effects of individual symptoms of gastroesophageal reflux disease co‐existing functional dyspepsia on patients' daily lives: A prospective, observational study

**DOI:** 10.1002/jgh3.12837

**Published:** 2022-11-05

**Authors:** Tatsuya Nakada, Kimio Isshi, Nobuyuki Matsuhashi, Katsuhiko Iwakiri, Takeshi Kamiya, Noriaki Manabe, Kazuhide Higuchi, Takashi Joh, Atsushi Oshio, Maiko Ogawa, Atsushi Hokari, Masayuki Saruta, Ken Haruma, Koji Nakada

**Affiliations:** ^1^ Division of Gastroenterology and Hepatology, Department of Internal Medicine, Katsushika Medical Center The Jikei University School of Medicine Tokyo Japan; ^2^ Isshi Gastro‐Intestinal Clinic and Department of Endoscopy The Jikei University School of Medicine Tokyo Japan; ^3^ Tokyo General Hospital, Gastroenterology Center Tokyo Japan; ^4^ Department of Gastroenterology Nippon Medical School Graduate School of Medicine Tokyo Japan; ^5^ Department of Medical Innovation Nagoya City University Graduate School Medical Sciences Nagoya Japan; ^6^ Division of Endoscopy and Ultrasonography, Department of Laboratory Medicine Kawasaki Medical School Okayama Japan; ^7^ Second Department of Internal Medicine Osaka Medical and Pharmaceutical University Osaka Japan; ^8^ Gamagori City Hospital Gamagori Japan; ^9^ Faculty of Letters, Arts and Sciences Waseda University Tokyo Japan; ^10^ Division of Gastroenterology and Hepatology, Department of Internal Medicine The Jikei University School of Medicine Tokyo Japan; ^11^ Department of General Internal Medicine 2 Kawasaki Medical School Kawasaki Hospital Okayama Japan; ^12^ Department of Laboratory Medicine The Jikei University School of Medicine Tokyo Japan

**Keywords:** functional dyspepsia, gastroesophageal reflux disease, multiple analysis, psychiatric disorder, quality of life

## Abstract

**Background and Aim:**

Patients with gastroesophageal reflux disease (GERD) frequently also have functional dyspepsia (FD) symptoms, which impair their quality of life. However, the magnitude and characteristics of the effects of each symptom on daily life have been unclarified. Using multiple regression analysis, we aimed to clarify these questions.

**Methods:**

We enrolled 290 patients from 29 institutions across Japan, in this prospective, observational study. Patients responded to three questionnaires (Gastroesophageal Reflux and Dyspepsia Therapeutic Efficacy and Satisfaction Test [GERD‐TEST], Hospital Anxiety and Depression Scale [HADS], and 8‐item Short‐Form Health Survey [SF‐8]) before and after 4 weeks of proton pump inhibitor treatment. Pearson correlation and multiple regression analyses were conducted between symptoms such as typical GERD, epigastric pain syndrome (EPS) and postprandial distress syndrome (PDS) of FD, and aspects of daily life, namely, level of satisfaction with the daily life of GERD‐TEST, anxiety and depression score of HADS, and physical and mental component summary of SF‐8.

**Results:**

Pearson correlation analysis showed a significant correlation in all combinations between GERD/FD‐EPS/FD‐PDS symptoms and the nine aspects of daily life. However, multiple regression analysis results deviated from these results, with the most significant effects seen in the PDS‐symptom subscale (SS) on the five aspects of daily life, that is, dissatisfaction with eating, daily life‐SS, anxiety, depression, and mental component summary (MCS) whereas the significant effects in GERD‐SS on five aspects of daily life, that is, dissatisfaction for eating, anxiety, depression, physical component summary, and MCS, disappeared.

**Conclusion:**

Dealing with co‐existing FD symptoms without overlooking them may be important in the management of GERD.

## Graphical abstract

Patients with gastroesophageal reflux disease (GERD) frequently also have co‐existing symptoms of functional dyspepsia (FD). The authors aimed to investigate the effects of each symptom of typical GERD, co‐existing FD epigastric pain syndrome (EPS), and postprandial distress syndrome (PDS) on patients’ level of satisfaction in daily life, the presence of psychiatric disorders, and quality of life using multiple regression analysis. Of the three symptoms, FD‐PDS symptom had the most prominent effect on the various aspects of daily life, whereas the impact of GERD symptom on daily life was rather modest.

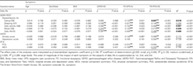

## Introduction

Gastroesophageal reflux disease (GERD) is common and frequently encountered in daily clinical practice. GERD symptoms are known to affect various aspects of daily life,^1^, ^2^, ^3^, ^4^ is associated with psychiatric disorders such as anxiety and depression,^4^, ^5^, ^6^, ^7^, ^8^ and can reduce a patient's quality of life (QOL).^3^, ^9^, ^10^, ^11^, ^12^, ^13^ GERD patients often have functional dyspepsia (FD) as well,^14^, ^15^, ^16^, ^17^ and it has been reported that FD symptoms concurrent with GERD significantly reduce QOL compared to GERD symptoms alone.^18^ However, the magnitude and characteristics of the effects of individual GERD and FD symptoms on various aspects of daily life have not been clarified. Therefore, we investigated the magnitude and characteristics of the effects of typical GERD, co‐existing FD epigastric pain syndrome (EPS), and postprandial distress syndrome (PDS) on patients' level of satisfaction in daily life, the presence of psychiatric disorders, and QOL.

## Patients and methods

### Study design

This was a multicenter, prospective, observational study conducted across 29 Japanese institutions, where one or more investigators per institution was a member of the Society for GERD, a Japanese collaborative research group consisting of GERD specialists. The study was conducted in accordance with the Declaration of Helsinki (sixth revision, 2008), after approval by either the relevant institution's ethics committee or the central ethics committee of Nishi Clinic, Osaka, Japan. The study was registered with the University Hospital Medical Information Network Center Clinical Trials Registry in Japan (reference number UMIN000006614). The data collection period of this study was from April 2011 to March 2012. Written informed consent was obtained from all enrolled patients.

### Patients

Outpatients with symptomatic GERD who received proton pump inhibitor (PPI) treatment during routine clinical care were recruited. After endoscopic examination, patients were treated with a PPI at a dosage approved in Japan before the start of this study (April 2011), that is, omeprazole 20 mg, lansoprazole 30 mg, or rabeprazole 10 or 20 mg once daily. Inclusion criteria were: (i) moderate or severe heartburn or acid regurgitation at least once a week or mild heartburn or acid regurgitation at least twice a week during the 2 weeks prior to the start of the study (Montreal definition);^19^ (ii) at least 20 years of age; and (iii) provision of written informed consent.

Exclusion criteria were (i) comorbidity or history of disease that could potentially affect the study results (e.g. Zollinger‐Ellison syndrome, inflammatory bowel disease, irritable bowel syndrome [IBS], esophageal stricture, eosinophilic esophagitis, achalasia, malabsorption, or cerebrovascular disease); (ii) concurrent symptoms of concern such as vomiting, peptic ulcer—except those in the scarred stage—and mental disorders; severe hepatic, renal, or cardiac diseases; uncontrolled metabolic diseases; neurological diseases; collagen diseases; or other diseases; (iii) confirmed or suspected malignancy; (iv) history of gastrointestinal tract resection or vagotomy; (v) history of hypersensitivity to PPIs or their excipients; (vi) Helicobacter pylori eradication within 6 months before enrollment; (vii) pregnancy, possible pregnancy, or breastfeeding; (viii) ingestion of PPI or histamine type2 (H2)‐receptor antagonist within 1 week of enrollment; and (ix) patients otherwise deemed to be ineligible by the attending physician. Prohibited concomitant drugs were those that might affect the study results (PPIs other than the study drugs, H2‐receptor antagonists, prokinetic agents, gastric mucosal protective agents, and anticholinergic drugs) and drugs that might interact with the study drugs.

### Assessments

Severity of reflux esophagitis was assessed according to the modified Los Angeles classification system.^20^ Patients' demographic and clinical characteristics were recorded before beginning PPI therapy (0 weeks) with a series of questionnaires. GERD and dyspeptic symptoms and dissatisfaction with daily life were assessed using the Gastroesophageal Reflux and Dyspepsia Therapeutic Efficacy and Satisfaction Test (GERD‐TEST)^3^ and the acute (1‐week‐recall) version of the 8‐item Short‐Form Health Survey (SF‐8),^21^ respectively, at 0, 2, and 4 weeks after PPI treatment. Severity of the psychiatric disorder was assessed using the Hospital Anxiety and Depression Scale (HADS)^22^ at 0 and 4 weeks. All questionnaires were completed and mailed to the data center by the study participants.

### Questionnaires for data collection

Patient characteristics were recorded using a questionnaire that included sex, age, height, weight, and lifestyle factors (regularity of daily life, consumption of caffeine‐containing beverages or high‐fat meals, smoking status, and alcohol consumption). The GERD‐TEST questionnaire is a patient‐reported questionnaire composed of 13 items for investigating GERD and dyspeptic symptoms, its impact on the patient's daily life, and patient's impression of the therapy. Questions (Q) 1 to 5 of the GERD‐TEST assess the severity of GERD and FD symptoms; Q6‐9 assess the impact of symptoms on daily life, including eating, sleeping, daily activity, and mood; Q10–12 evaluate the therapeutic response to the PPIs; Q13 asks about medication compliance; Q1–11 and Q13 use a Likert scale; while Q12 uses a numeric rating scale (NRS) (Table 1). The HADS is a well‐established scale that measures the severity of psychiatric disorders such as anxiety and depression, with each subscale composed of seven items. The SF‐8 is a generic questionnaire used to investigate health status and is composed of a physical component summary (PCS) and a mental component summary (MCS). These scores are normalized to the general population, with higher scores indicating better physical and mental QOL, with a normative score of 50 and a standard deviation of 10.

**Table 1:** Gastroesophageal reflux and dyspepsia therapeutic efficacy and satisfaction test (GERD‐TEST)

Q1. Have you been bothered by heartburn during the past week? (By heartburn we mean a burning pain or discomfort behind the breastbone in your chest)										
Q2. Have you been bothered by acid regurgitation during the past week? (By acid regurgitation we mean regurgitation or flow of sour or bitter fluid into your mouth)										
Q3. Have you been bothered by epigastric pain or burning during the past week? (Epigastric pain includes any type of pain in the stomach)										
Q4. Have you been bothered by postprandial fullness during the past week? (Postprandial fullness refers to discomfort or a sensation of heaviness caused by the food you consume remaining in the stomach)										
Q5. Have you been bothered by early satiation during the past week? (Early satiation refers to the inability to finish a normal‐sized meal)										
Response scale for Q1–Q5:										
1 = no discomfort at all, 2 = slight discomfort, 3 = mild discomfort, 4 = moderate discomfort, 5 = moderately severe discomfort, 6 = severe discomfort, 7 = very severe discomfort										
Q6. During the past week, how often have you felt dissatisfaction because you were unable to eat meals as you intended due to chest and stomach symptoms? (Not being able to eat as you intended refers to the inability to eat the sufficient amount of food you want to eat at an uninhibited, natural pace)										
Q7. During the past week, how often have you felt dissatisfaction due to impaired sleep caused by chest and stomach symptoms?										
Q8. During the past week, how often have you felt dissatisfaction due to impairment of your work, housework, or other daily activities caused by chest and stomach symptoms?										
Q9. During the past week, how often have you felt dissatisfaction because you were in a bad mood due to chest and stomach symptoms?										
Response scale for Q6–Q9										
1 = not at all, 2 = slightly, 3 = moderately, 4 = quite a lot, 5 = extremely										
Q10. During the past week, how often have you wanted another drug in addition to the drug your doctor prescribed because of intense symptoms of heartburn and acid regurgitation?										
1 = not at all, 2 = on 1 day, 3 = on 2 to 3 days, 4 = on 4 to 5 days, 5 = always										
Q11. During the past week, how have you felt about symptoms of heartburn and acid regurgitation as compared with the symptom severity before the current treatment?										
1 = extremely improved, 2 = improved, 3 = slightly improved, 4 = not changed, 5 = aggravated										
Q12. If 10 corresponds to your symptoms before the current treatment and 0 is “symptom‐free,” what number corresponds to symptoms of heartburn and acid regurgitation during the past week? Please circle the applicable score below:										
0	1	2	3	4	5	6	7	8	9	10
Symptom‐free				Symptoms before current treatment						
Q13.What proportion of the proton pump inhibitor prescribed to you did you take as instructed?										
1 = took drug as instructed, 2 = generally took drug as instructed (took at least three‐quarters of the drug prescribed), 3 = sometimes forgot (took at least half but less than three‐quarters of the drug prescribed, 4 = took little (took less than half of the drug prescribed), 5 = did not take any.										

Before therapy, questions about treatment efficacy and adherence (Q10–Q13) were excluded. The following scores were defined: GERD symptom score = (Q1 + Q2)/2, Epigastric pain/burning symptoms score = Q3, Postprandial distress symptom subscale = (Q4 + Q5)/2, Residual symptom rate (%) = 100 × (GERD symptom score at 4 weeks − 1)/(GERD symptom score at 0 week − 1).

### Definitions of subscale scores in GERD‐TEST

The GERD‐symptom subscale (SS) was defined as the mean score for heartburn (Q1) and regurgitation (Q2). The FD‐EPS‐Sx (symptom) was defined as scores for epigastric pain/burning (Q3) and the FD‐PDS‐SS was defined as the mean of scores for postprandial fullness (Q4) and early satiation (Q5). The dissatisfaction with daily life‐SS was defined as the mean of scores for dissatisfaction with eating (Q6), sleeping (Q7), daily activities (Q8), and mood (Q9).

### Outcome measures

We set the followings which were measured before treatment as evaluation items in this study: GERD‐SS, FD‐EPS‐Sx, and FD‐PDS‐SS; which express the severity of each symptom of GERD and FD, dissatisfaction with eating, sleeping, daily activity, mood, and daily life‐SS; which express the patients' level of satisfaction for daily life, HADS anxiety and depression; and the SF‐8 PCS and MCS. We then investigated the correlation between the GERD‐SS, FD‐EPS‐Sx, and FD‐PDS‐SS symptoms and the various aspects of daily life (i.e. level of satisfaction for daily life, psychiatric disorders, and QOL) as well as the effect magnitude of these symptoms.

### Statistical analysis

Pearson correlation analysis was performed to examine the correlation between symptoms (GERD‐SS, FD‐EPS‐Sx, FD‐PDS‐SS) and daily life (the level of satisfaction for daily life [eating, sleeping, daily activities, mood, daily life‐SS], HADS anxiety and depressive score, PCS and MCS of SF‐8). In addition, we added age, sex, and BMI as explanatory variables to perform multiple regression analysis to identify the effect of individual symptoms on the above various aspects of daily life. The effect sizes of the analyses were interpreted as *r* (Pearson correlation coefficient) and *β* (standardized regression coefficient) ≧0.100, *R* ^2^ (coefficient of determination) ≧0.020: small; *r* and *β* ≧ 0.300, *R* ^2^ ≧ 0.130: medium; and *r* and *β* ≧ 0.500, *R* ^2^ ≧ 0.260: large. Data analysis was undertaken using JMP12.0.1 software (SAS Institute Inc., Cary, NC, USA). The values of *P* < 0.05 were considered significant.

## Results

### Patients' characteristics

We enrolled 290 patients who had GERD symptoms that satisfied the Montreal definition, satisfied the criteria of the present study, underwent endoscopy prior to the start of PPI administration, and responded to the GERD‐TEST/HADS/SF‐8. Patient demographics were as follows: average age, 57.5 ± 13.9 years; average BMI, 24 ± 3.9 kg/m^2^; and 178 males (61%). The severity of esophageal mucosal injury by endoscopy was as follows: non‐erosive reflux disease (NERD), 107 patients (37%); and erosive reflux disease (ERD), 183 patients (63%), with more ERD patients (Table 2).

**Table 2:** Patients' characteristics (*n* = 290)

Age (year)^†^	57.5 ± 13.9
BMI (kg/m^2^)	24.0 ± 3.9
Sex: *n* (%)	
Male	178 (61)
Female	112 (39)
Endoscopic finding: *n* (%)	
LA classification	
NERD	107 (37)
Grade N	62 (21)
Grade M	45 (16)
ERD	183 (63)
Grade A	94 (32)
Grade B	60 (21)
Grade C	21 (7)
Grade D	8 (3)

^†^ Data are presented as mean ± SD.

BMI, body mass index; ERD, erosive reflux disease; LA, Los Angeles; NERD, non‐erosive reflux disease.

### The scores for GERD‐TEST, HADS and SF‐8

The symptom scores were, in descending order: GERD‐SS, 3.4 ± 1.2; FD‐EPS‐Sx, 3.1 ± 1.4; and FD‐PDS‐SS, 2.7 ± 1.2. The scores for level of satisfaction for daily life were as follows: dissatisfaction with eating, 2.0 ± 1.1; sleeping, 2.1 ± 1.1; daily activity, 2.0 ± 1.0; mood, 2.6 ± 1.1; and daily‐life‐SS, 2.2 ± 0.8. The HADS anxiety score was 6.3 ± 3.7, and the depression score was 5.5 ± 3.8. The SF‐8 PCS was 45.9 ± 6.8, and MCS was 46.9 ± 7.0 (Table 3).

**Table 3:** The scores for GERD‐TEST, HADS, and SF‐8 (*n* = 290)

GERD‐TEST^†^	
GERD‐SS (Q1, Q2)	3.4 ± 1.2
FD‐EPS‐Sx (Q3)	3.1 ± 1.4
FD‐PDS‐SS (Q4, Q5)	2.7 ± 1.2
Dissatisfaction for	
Eating (Q6)	2.0 ± 1.1
Sleeping (Q7)	2.1 ± 1.1
Daily activity (Q8)	2.0 ± 1.0
Mood (Q9)	2.6 ± 1.1
Daily life‐SS	2.2 ± 0.8
HADS^†^	
Anxiety score	6.3 ± 3.7
Depression score	5.5 ± 3.8
SF‐8^†^	
PCS	45.9 ± 6.8
MCS	46.9 ± 7.0

^†^ Data are presented as mean ± SD.

GERD+FD symptoms 219 (75.5%), GERD+FD‐EPS symptoms 39(13.5%), GERD+FD‐PDS symptoms 34(11.7%), GERD+FD‐EPS+FD‐PDS symptom 146(50.3%).

EPS, epigastric pain syndrome; FD, functional dyspepsia; GERD, gastroesophageal reflux disease; GERD‐TEST, Gastroesophageal Reflux and Dyspepsia Therapeutic Efficacy and Satisfaction Test; HADS, Hospital anxiety and depression scale; MCS, mental component summary; PCS, physical component summary; PDS, postprandial distress syndrome; SF‐8, The 8‐item Short‐Form Health Survey; SS, symptom subscale; Sx, symptom.

### The co‐existence rate of FD symptom

When setting cases where patients had three or more points in the GERD‐TEST as symptoms being “present,” for Q1 (heartburn), Q2 (acid regurgitation), Q3 (epigastric pain/burning), Q4 (postprandial fullness), or Q5 (early satiation), results showed that there were 219 patients (75.5%) with the co‐existence of GERD symptoms (Q1 and/or Q2) and FD symptoms (Q3 and/or Q4 and/or Q5). The breakdown was as follows: co‐existence with EPS symptoms, 39 patients (13.5%); co‐existence with PDS symptoms (11.7%); and co‐existence with both symptoms, 146 patients (50.3%) (Table 3).

### Correlations between individual GERD/FD symptoms and various aspects of daily life (Pearson correlation analysis)

There was a significant correlation in all combinations between each symptom score of GERD‐SS, FD‐EPS‐Sx, and FD‐PDS‐SS and various aspects of daily life, or in other words, the level of satisfaction for daily life (dissatisfaction with eating, sleeping, daily activity, mood, daily life‐SS), HADS anxiety and depression, and SF‐8 PCS and MCS (*P* < 0.001) (Table 4).

**Table 4:** Correlations between individual GERD/FD symptoms and various aspects of daily life (Pearson correlation analysis)

	Symptoms					
Questionnaires	GERD‐SS		FD‐EPS‐Sx		FD‐PDS‐SS	
Daily life	*r*	*P*‐value	*r*	*P*‐value	*r*	*P*‐value
GERD‐TEST						
Dissatisfaction for						
Eating (Q6)	0.393	<0.001	0.452	<0.001	**0.708**	<0.001
Sleeping (Q7)	0.457	<0.001	0.445	<0.001	0.364	<0.001
Daily activity (Q8)	**0.517**	<0.001	0.483	<0.001	0.495	<0.001
Mood (Q9)	**0.585**	<0.001	**0.520**	<0.001	**0.544**	<0.001
Daily life‐SS	**0.614**	<0.001	**0.595**	<0.001	**0.655**	<0.001
HADS						
Anxiety score	0.305	<0.001	0.339	<0.001	0.386	<0.001
Depression score	0.256	<0.001	0.274	<0.001	0.323	<0.001
SF‐8						
PCS	−0.266	<0.001	−0.311	<0.001	−0.218	<0.001
MCS	−0.238	<0.001	−0.327	<0.001	−0.421	<0.001

The effect sizes of the analyses were interpreted as *r* (Pearson correlation coefficient) ≧ 0.100: small, *r* ≧ 0.300: medium (underlined), and *r* ≧ 0.500: large (bold).

EPS, epigastric pain syndrome; FD, functional dyspepsia; GERD, gastroesophageal reflux disease; GERD‐TEST, Gastroesophageal Reflux and Dyspepsia Therapeutic Efficacy and Satisfaction Test; HADS, hospital anxiety and depression scale; MCS, mental component summary; PCS, physical component summary; PDS, postprandial distress syndrome; SF‐8, The 8‐item Short‐Form Health Survey; SS, symptom subscale; Sx, symptom.

### Effects of individual GERD/FD symptoms on various aspects of daily life (multiple regression analysis)

Multiple regression analysis results greatly deviated from the Pearson correlation analysis results. For GERD symptoms, five out of nine various aspects of daily life (dissatisfaction with eating, anxiety and depression, PCS, and MCS) were no longer significantly affected. For FD‐EPS symptoms, depression was no longer significantly affected; and for FD‐PDS symptoms, PCS was no longer significantly affected. Additionally, there was a diminished magnitude of the effect of each symptom in the multiple variable aspects of daily life. Age, sex, and BMI had no significant effect on various aspects of daily life, except that PCS was significantly better in males.

The items for the level of satisfaction for daily life that was significantly affected by the three symptoms, in descending order of effect: dissatisfaction for eating; FD‐PDS‐SS, and FD‐EPS‐Sx, sleeping; GERD‐SS, FD‐EPS‐Sx, and FD‐PDS‐SS, daily activity; GERD‐SS, FD‐PDS‐SS, and FD‐EPS‐Sx, mood; GERD‐SS, FD‐PDS‐SS, and FD‐EPS‐Sx, and daily life‐SS; FD‐PDS‐SS, GERD‐SS, and FD‐EPS‐Sx, respectively (Table 5).

**Table 5:** Effects of background factors and individual GERD/FD symptoms on various aspects of daily life (multiple regression analysis)

	Symptoms													
Questionnaires	Age		Sex(Male)		BMI		GERD‐SS		FD‐EPS‐Sx		FD‐PDS‐SS		*R* ^2^	*P*‐value
Daily life	*β*	*P*‐value	*β*	*P*‐value	*β*	*P*‐value	*β*	*P*‐value	*β*	*P*‐value	*β*	*P*‐value		
GERD‐TEST														
Dissatisfaction for														
Eating (Q6)	0.014	0.756	‐0.033	0.462	−0.076	0.082	0.000	0.998	0.133^**2nd**^	0.021	**0.637** ^**1st**^	<0.001	**0.519**	<0.001
Sleeping (Q7)	0.085	0.119	0.051	0.356	−0.056	0.300	0.256^**1st**^	<0.001	0.221^**2nd**^	0.002	0.146^**3rd**^	0.023	0.256	<0.001
Daily activity (Q8)	−0.046	0.369	−0.036	0.493	−0.067	0.185	0.282^**1st**^	<0.001	0.152^**3rd**^	0.023	0.258^**2nd**^	<0.001	**0.357**	<0.001
Mood (Q9)	−0.064	0.178	− 0.073	0.134	−0.051	0.279	0.344 ^**1st**^	<0.001	0.133^**3rd**^	0.032	0.273^**2nd**^	<0.001	**0.440**	<0.001
Daily life‐SS	−0.004	0.931	−0.024	0.577	−0.075	0.071	0.281^**2nd**^	<0.001	0.200^**3rd**^	<0.001	0.408 ^**1st**^	<0.001	**0.560**	<0.001
HADS														
Anxiety score	−0.033	0.568	−0.019	0.756	−0.080	0.169	0.074	0.326	0.140^**2nd**^	0.067	0.258^**1st**^	<0.001	0.174	<0.001
Depression score	0.038	0.525	0.071	0.249	0.026	0.659	0.077	0.322	0.105	0.181	0.254^**1st**^	<0.001	0.128	<0.001
SF‐8														
PCS	−0.107	0.075	0.168	0.006	−0.011	0.852	−0.090	0.249	−0.214^**1st**^	0.007	−0.044	0.534	0.143	<0.001
MCS	0.069	0.235	0.050	0.400	0.053	0.358	0.060	0.431	−0.169^**2nd**^	0.029	−0.329 ^**1st**^	<0.001	0.195	<0.001

The effect sizes of the analyses were interpreted as *β* (standardized regression coefficient) ≧ 0.100, *R* ^2^ (coefficient of determination) ≧0.020: small, *β* ≧ 0.300, *R* ^2^ ≧ 0.130: medium (underlined), *β* ≧ 0.500, *R* ^2^ ≧ 0.260: large (bold). The order of magnitude of the impact of each symptom on the aspects of daily life is superscripted as 1st, 2nd, and 3rd.

BMI, Body mass index; EPS, epigastric pain syndrome; FD, functional dyspepsia; GERD, gastroesophageal reflux disease; GERD‐TEST, Gastroesophageal Reflux and Dyspepsia Therapeutic Efficacy and Satisfaction Test; HADS, hospital anxiety and depression scale; MCS, mental component summary; PCS, physical component summary; PDS, postprandial distress syndrome; SF‐8, The 8‐item Short‐Form Health Survey; SS, symptom subscale; Sx, symptom.

The items for which psychiatric disorders were significantly affected by the three symptoms, in descending order of effect: anxiety; FD‐PDS‐SS, and FD‐EPS‐Sx, and depression; FD‐PDS‐SS, respectively (Table 5).

The QOL items that were significantly affected by the three symptoms, in descending order of effect: PCS; FD‐EPS‐Sx, and MCS; FD‐PDS‐SS, and FD‐EPS‐Sx, respectively (Table 5).

Of the three symptoms, FD‐PDS had the most prominent effect, with the most significant effects on five out of nine various aspects of daily life (i.e. dissatisfaction with eating, daily life‐SS, anxiety and depression, and MCS).

## Discussion

GERD is known to be highly associated with FD,^14^, ^15^, ^16^, ^17^ both GERD and FD symptoms have been reported to interfere with daily activities and are also associated with psychiatric disorders, which reduce the patients' QOL.^1^, ^2^, ^3^, ^4^, ^5^, ^6^, ^7^, ^8^, ^9^, ^10^, ^11^, ^12^, ^13^, ^23^, ^24^, ^25^, ^26^ However, the exact manner in which individual symptoms of GERD and FD affect various aspects of patients' daily life has not yet been clarified. Therefore, we investigated the relationship between each symptom and various aspects of daily life. Pearson correlation analysis showed a significant correlation in all combinations between each symptom and nine items that constitute the living conditions. However, multiple regression analysis showed that the effect of GERD symptoms on daily life was not significant for five out of nine items. Co‐existing FD symptoms had a more significant effect on the various living conditions; particularly, FD‐PDS‐SS had the greatest effects on dissatisfaction with eating, psychiatric disorders, and mental health. This paper is the first to use multiple regression analysis to clarify the effect of each individual symptom on daily life when FD symptoms co‐exist with GERD.

The prevalence of GERD in Japan has significantly increased since the latter half of the 1990 s,^27^, ^28^ which has been attributed to changes in lifestyles, westernization of dietary habits, and a decrease in the infection rate of *Helicobacter pylori*.^29^ GERD symptoms interfere with various aspects of daily life (diet, sleep, daily activity, mood). There have been multiple reports on the association between GERD and sleep disorders,^1^, ^2^, ^30^, ^31^ and it is reported that the frequency of sleep disorders was high in patients with heartburn symptoms—regardless of the presence or severity of esophageal mucosal disorders.^2^ Additionally, GERD symptoms cause physical and mental disorders, affecting work and social activities,^4^ and are also associated with psychiatric disorders such as anxiety and depression,^3^, ^4^, ^5^, ^6^, ^7^, ^8^ resulting in a decrease in QOL.^9^, ^10^, ^11^, ^12^, ^13^

The co‐existence of FD has been reported to be common in GERD patients, with an incidence between 9–63.8%.^17^, ^32^, ^33^, ^34^, ^35^ FD symptoms also affect various aspects of daily life, reducing the patient's QOL,^23^, ^24^, ^25^, ^26^ and it has been reported that the co‐existence of FD and GERD symptoms caused a greater decrease in the patients' QOL than GERD symptoms alone.^18^ However, the magnitude and characteristics of the effects of co‐existing GERD and FD symptoms on each item that represents the various aspects of daily life have not yet been clarified.

The GERD‐TEST has been developed and validated by the study committee of the GERD Society^3^ as a simple and easy‐to‐understand questionnaire with a minimum number of items and is one of its official questionnaires. In this study, we used three questionnaires (GERD‐TEST,^3^ HADS,^22^ SF‐8^21^) simultaneously, and used Pearson correlation analysis and multiple regression analysis in order to investigate the correlation and magnitude of effects between the individual GERD/FD‐EPS/FD‐PDS symptoms and various aspects of daily life. Our Pearson correlation results showed significant correlations in all combinations of the three symptoms and nine various aspects of daily life. However, multiple regression analysis results greatly deviated from Pearson correlation analysis results: for GERD‐SS, five aspects of daily life were no longer significantly affected by symptoms; whereas for FD‐EPS‐Sx and FD‐PDS‐SS, one aspect of daily life was no longer significantly affected by symptoms. Furthermore, the magnitude of the effect decreased for several items. There have been several reports on the effects of GERD and co‐existing FD symptoms on daily life,^36^, ^37^ their association with psychiatric disorders,^37^, ^38^, ^39^, ^40^ and reduction in QOL,^18^, ^32^, ^40^, ^41^ but these studies did not adjust for the effects of GERD or co‐existing FD using multiple analysis; thus, the effects of symptoms may be overestimated, and caution is required in their interpretation.

Besides symptoms, background factors such as age, gender, and BMI may also affect various aspects of daily life. Therefore, age, sex, and BMI were also added to the three symptoms as explanatory variables for multiple regression analysis. As a result, these background factors had little effect on daily life (Table 5).

In the future, investigations of the effects of gastrointestinal symptoms on daily life should also incorporate co‐existing symptoms and examine the effects of each symptom using multiple analyses. Patients who complained of GERD symptoms reported adverse effects on various aspects of their daily life, but multiple regression analysis results showed that the co‐existing FD symptoms—rather than GERD symptoms—had a noticeable effect that could not be ignored. Particularly, FD‐PDS‐SS had a large effect on dissatisfaction with eating, psychiatric disorders such as anxiety and depression, and the degree of mental health in SF‐8.

A limitation of this paper is that it only investigated the effects of typical symptoms of GERD and FD on daily life. It has been suggested that atypical symptoms of GERD and FD have a considerable effect on the daily life of patients;^42^ thus, prospective clinical studies should investigate the effects of GERD and FD on daily life, including not only typical but also atypical co‐existing symptoms.

We showed that co‐existing FD symptoms have a significant effect on various aspects of the daily life of GERD patients. It is postulated that investigating not only the typical GERD symptoms, but also the co‐existing FD symptoms, and understanding the magnitude and characteristics of each symptom in various aspects of daily life, would be useful in estimating the burden in the patient's daily life and mental health. This could provide more appropriate treatment options in the management of GERD.

## Data Availability

The data that support the findings of this study are available from the corresponding author, Tatsuya Nakada, upon reasonable request.
